# Up-Regulated FASN Expression Promotes Transcoelomic Metastasis of Ovarian Cancer Cell through Epithelial-Mesenchymal Transition

**DOI:** 10.3390/ijms150711539

**Published:** 2014-06-27

**Authors:** Li Jiang, Hong Wang, Jiarui Li, Xuhong Fang, Hong Pan, Xiangliang Yuan, Ping Zhang

**Affiliations:** 1 Department of Gynecology, Xin Hua Hospital Affiliated to Shanghai Jiao Tong University School of Medicine, 1665 Kongjiang Road, Shanghai 200092, China; E-Mails: jianglixinhua@gmail.com (L.J.); mms2002@126.com (H.W.); ljr-xh@hotmail.com (J.L.); fangxuhongxh@126.com (X.F.); panhong1978@hotmail.com (H.P.); 2 Department of Clinical Laboratory, Xin Hua Hospital Affiliated to Shanghai Jiao Tong University School of Medicine, 1665 Kongjiang Road, Shanghai 200092, China

**Keywords:** ovarian cancer, fatty acid synthase, tumorigenicity, EMT

## Abstract

Fatty acid synthase (FASN), responsible for the *de novo* synthesis of fatty acids, has been shown to act as an oncogene in various human cancers. However, the mechanisms by which FASN favors the progression of ovarian carcinoma remain unknown. In this study, we evaluated FASN expression in ovarian cancer and investigated how FASN regulates the aggressiveness of ovarian cancer cells. Our results show that increased FASN is associated with the peritoneal metastasis of ovarian cancers. Over-expression of FASN results in a significant increase of tumor burden in peritoneal dissemination, accompanied by augment in cellular colony formation and metastatic ability. Correspondingly, FASN knockdown using RNA interference in ovarian cancer cells inhibits the migration *in vitro* and experimental peritoneal dissemination *in vivo*. Mechanistic studies reveal that FASN promotes Epithelial-mesenchymal Transition (EMT) via a transcriptional regulation of *E*-cadherin and *N*-cadherin, which is also confirmed by luciferase promoter activity analysis. Taken together, our work demonstrates that FASN promotes the peritoneal dissemination of ovarian cancer cells, at least in part through the induction of EMT. These findings suggest that FASN plays a critical role in the peritoneal metastasis of ovarian cancer. Targeting *de novo* lipogenesis may have a therapeutic potential for advanced ovarian cancer.

## 1. Introduction

Ovarian cancer is the second most common gynecologic malignancy and the most common cause of gynecologic cancer related death worldwide [1]. Approximately 70% of women with ovarian cancer are diagnosed at an advanced stage with metastases, and only 45% of patients with advanced stage ovarian cancer survive 5 years after initial diagnosis [2]. The biological behavior of ovarian carcinoma is unique and different from the classic pattern of hematogenous metastasis found in most other cancers. Usually, patients with ovarian carcinoma have locally advanced disease with contiguous extension to the outside of ovaries and abdominal peritoneum [3,4]. Transcoelomic metastasis, the most common route, is responsible for the greatest morbidity and mortality in women with this disease [5]. Unfortunately, little is known about the mechanisms behind this process. Thus, it is necessary to improve our understanding of how ovarian carcinoma spreads to the peritoneum.

The disruption of signaling networks including multiple alterations in the oncogenic pathways and significant changes in cellular metabolism leads to the progression of cancer to a metastatic phenotype [6]. Most normal human tissues acquire the majority of required fatty acids from the circulation, which results in extremely low original lipogenesis and expression of lipogenic enzymes. In contrast, most of the fatty acids in malignant cells are derived from *de novo* lipogenesis due to increased endogenous lipid biosynthesis during malignant transformation [7]. Various tumors and their precursor lesions unexpectedly undergo exacerbated endogenous fatty acids biosynthesis irrespective of the levels of extracellular lipids [8]. The increased lipogenesis is represented by significantly elevated expression and hyperactivity of numerous lipogenic enzymes. Fatty acid synthase (FASN) is the main enzyme involved in fatty acids synthesis that catalyses the NADPH-dependent condensation of acetyl-coenzyme A (CoA) and malonyl-CoA to produce palmitate [9].

Recent evidence shows that during *de novo* fatty acid synthesis, fatty acid synthase plays a crucial role in the carcinogenesis process of various cancers [6,10,11,12,13,14,15]. Increased expression of FASN was shown in tumors and correlated with decreased survival and increased disease recurrence in several tumor types. Furthermore, increased FASN expression has a positive relation with tumor aggressiveness and a poor prognosis for renal cell carcinoma [16]. The overexpression of FASN promots the colorectal tumorigenesis *in vivo* [17]. Functional assays by RNA interference provide adequate evidence to link the oncogenic role of FASN between activation of lipogenesis and carcinogenesis [17,18,19]. It has also been shown that fatty acid metabolism contributes to ovarian cancer tumorigenesis [20]. However, the impact of aberrant expression of FASN on metastases remains in infancy and the detailed mechanism underlying the metastasis of ovarian cancer needs to be elucidated.

To study the role of FASN in the metastases of human ovarian cancer, we investigated the expression of FASN in human ovarian cancer tissue samples, as well as the function of FASN in human ovarian cancer cell lines. In this study, we demonstrate that elevated FASN expression promotes the malignant properties of ovarian cancer cells *in vitro*. Knockdown FASN by siRNA significantly suppresses experimental peritoneal metastasis of ovarian cancer.

## 2. Results and Discussion

### 2.1. Results

#### 2.1.1. Increased Expression of Fatty Acid Synthase (FASN) Correlated with the Progression and Peritoneal Metastasis of Human Ovarian Cancer

**Figure 1:**
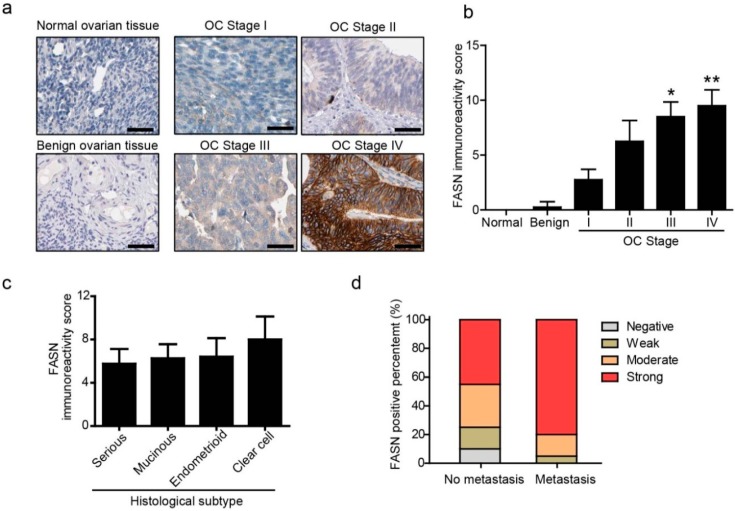
Over-expression of fatty acid synthase (FASN) was correlated with stage and peritoneal metastasis in ovarian cancer. (**a**) Representative images of FASN expression in normal ovarian tissue, benign ovarian tissue and primary ovarian cancer tissues (FIGO I, II, III, and IV) are shown (200× magnificaiton). Scale bar, 50 µm; (**b**) FASN is significantly increased in patients with ovarian cancer FIGO III and IV compare with FIGO I (*, *p* < 0.05; **, *p* < 0.01); (**c**) Immunoreactivity scores of FASN in different pathological subgroups are determined according to a semiquantitative method, as described in Materials and Methods; (**d**) FASN scores are significantly higher in peritoneal metastasis ovarian cancer than in non-metastasis specimens (*p* < 0.05).

To examine the potential clinical relevance of FASN to ovarian cancer progression, we sought to investigate FASN expression and its association with different clinicopathological parameters using a human ovarian cancer tissue microarray derived from patients with progressive ovarian disease. Normal ovarian tissue and benign ovarian tissue displayed undetectable to very low FASN staining whereas ovarian carcinomas showed FASN immunoreactivity in most cases (Figure 1a). The FASN staining score of high-grade carcinomas is significantly higher than that of low-grade carcinomas. Immunohistochemical staining for FASN revealed a correlation between FASN expression levels and ovarian cancer progression (Figure 1b). Next, we investigated whether FASN expression is correlated with key clinical parameters in human ovarian cancer. There were no significant correlations between high FASN expression and patient age, histological subtypes (Figure 1c), however, FASN expression was associated with FIGO stage (*p* < 0.05) (Figure 1b). Peritoneal transcoelomic dissemination is the main common metastasis causing the greatest morbidity and mortality in women with this disease, and we next determined the association of FASN expression with peritoneal metastasis. Statistical evaluation of immunoreactivity scores showed that expression of FASN was significantly unregulated in the patients with peritoneal metastasis compared with non-metastasis patients (Figure 1d). Taken together, these data strongly indicate that enhanced FASN may play a role in the progression of the primary ovarian cancer cell to peritoneal metastasis.

#### 2.1.2. FASN Promoted the Colony Formation of Ovarian Cancer Cell

**Figure 2:**
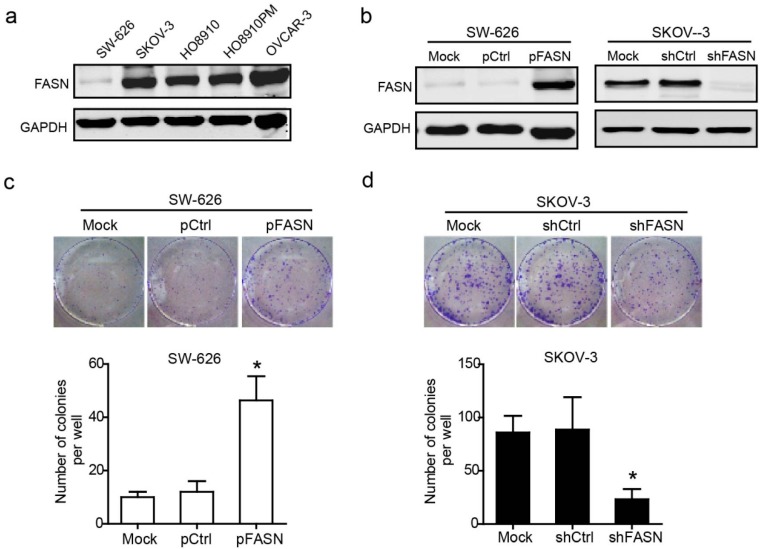
FASN increases the anchorage-independent growth of the ovarian cancer cell. (**a**) FASN expression in ovarian cancer cell lines was conducted by Western blot analysis; (**b**) SW-626 cell was transfected with pcDNA3.1 control vector (pCtrl) or FASN-pcDNA3.1 vector (pFASN), and confirmed by Western blot analysis. SKOV-3 was transfected with shRNA control vector (shCtrl) or shFASN vector (shFASN), and confirmed by Western blot analysis. Mock means transfection reagent only; (**c**) Over-expression of FASN increased a significant number of colonies formed on forced expression of FASN in SW-626 cell (*, *p* < 0.05); (**d**) Silencing of FASN by RNAi reduced the number of colonies formed on knock-down FASN in SKOV-3 cell (*, *p* < 0.05).

Given the correlation of FASN expression with clinically relevant features, we investigated FASN as a potential regulator of ovarian cancer cell biological characteristics. Firstly, we tested expression of FASN in a panel of ovarian cancer cell lines. Consistent with results obtained from human tissue arrays, western blot analysis showed high expression of FASN in most ovarian cancer cells (Figure 2a). Secondly, to examine how FASN affects the biology of ovarian cancer cells, we stably transduced SW-626 cell with an empty vector or FASN plasmid vector, and transduced SKOV-3 cell with control shRNA vector or shFASN vector to generate stable cells. We effectively over-expressed FASN in SW-626 cell and knock-down FASN in SKOV-3 cell (Figure 2b). SW-626 (control and FASN over-expression) and SKOV-3 cell (control and FASN knockdown) were tested for their ability to grow using colony formation assay. We observed a significant increase in the number of colonies formed when FASN was over-expressed in SW-626 cell (Figure 2c). Conversely, knock-down FASN decreased the number of colonies formed in SKOV-3 cell (Figure 2d).

#### 2.1.3. Constitute Expression of FASN Regulated the Motility and Invasive Capabilities of the Ovarian Cancer Cell

**Figure 3:**
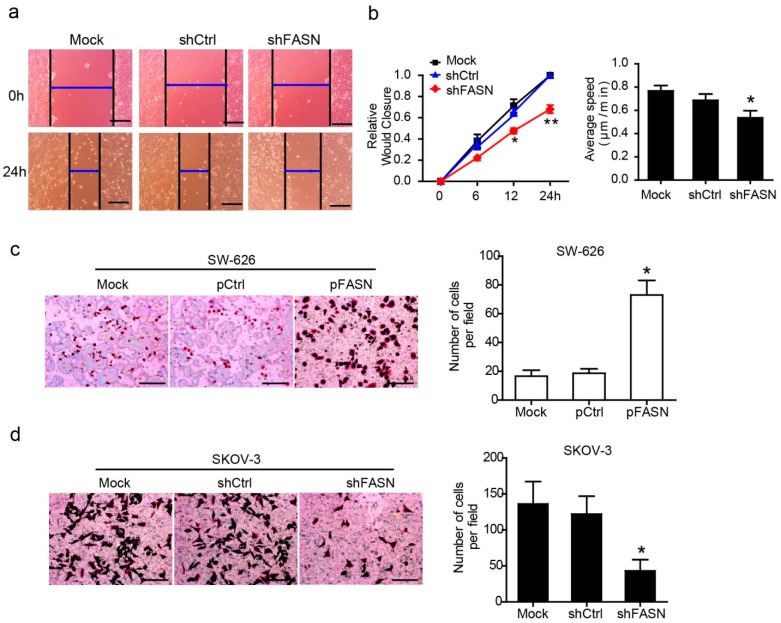
FASN increases the motility and metastasis of ovarian cancer cells. (**a**) For wound healing assay, FASN-knock down stable SKOV-3 cells were wounded by scratching and monitored over 24 h to determine the rate of wound closure (40× magnification); Scale bar, 200 µm; (**b**) Cell migration was assessed by measuring relative wound closure and average speed. Data represent mean ± SEM (**, *p* < 0.01; *, *p* < 0.05); (**c**) The metastatic properties of the SW-626 cell stable transfected with pFASN. Three independent experiments with three fields for each were carried out and representative fields were shown (*, *p* < 0.05) (400× magnification); Scale bar, 100 µm; (**d**) The metastatic properties of the SKOV-3 cell stable transfected with shFASN. The representative fields were shown (*, *p* < 0.05) (400× magnification). Scale bar, 100 µm.

Migration and invasion are critical steps in the initial progression of cancer that facilitate metastasis. To elucidate the association of FASN expression and ovarian cancer cell metastasis, we chose to analyze migration using two independent assays, monolayer wound healing and metastasis transwell assay. We first confirmed that overexpression or knockdown of FASN did not affect ovarian cancer cell viability in 24 h. In monolayer migration assays, wound closure of cells with FASN knockdown was modestly inhibited relative to non-target control shRNA-transfected cells (Figure 3a,b). Next, we analyzed directional migration toward fetal bovine serum (FBS) in transwell migration assays, which is a more relevant assay than wound healing for assessing metastatic potential. Over-expression of FASN significantly increased the migration of SW-626 cell (Figure 3c). Conversely, knockdown of FASN attenuated the migration of SKOV-3 cell (Figure 3d).

#### 2.1.4. FASN Leads to Peritoneal Implanting Metastases of Ovarian Cancer Cell *in Vivo*

**Figure 4:**
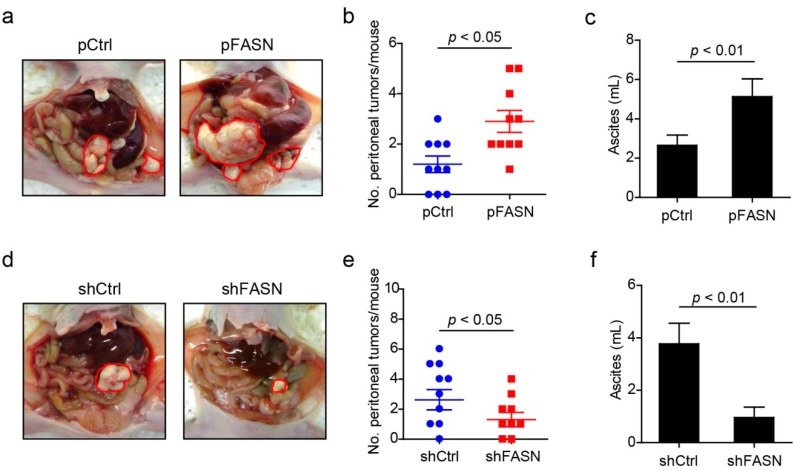
FASN promotes the peritoneal implantation of ovarian cancer cells. (**a**) Representative macroscopic peritoneal images of SW-626 cell stable transfected with control vector (pCtrl) or FASN vector (pFASN) are shown. Red circles indicate the tumor mass; (**b**) Scatter dot plots are shown for the numbers of peritoneal tumors per mouse implanted SW-626 cell stable transfected with control vector (pCtrl) or FASN vector (pFASN). Data represent mean ± SEM (*n* = 10); (**c**) Box plots are shown for peritoneal ascites per mouse implanted SW-626 cell stable transfected with control vector (pCtrl) or FASN vector (pFASN). Data represent mean ± SEM (*n* = 10); (**d**) Representative macroscopic peritoneal images of SKOV-3 cell stable transfected with control shRNA vector (shCtrl) or shFASN vector (shFASN) are shown. Red circles indicate the tumor mass; (**e**) Numbers of peritoneal tumor per mouse are calculated in a scatter dot plot; (**f**) The amounts of peritoneal ascites in mouse implanted with SKOV-3 cell stable transfected with shCtrl or shFASN are shown. Data represent mean ± SEM (*n* = 10).

To assess the role of FASN in ovarian cancer metastasis *in vivo*, we utilized an intraperitoneal xenograft mouse model. The extensive tumor development and peritoneal metastasis observed in mouse with overexpression FASN treatment was significantly increased in mice treated with control (Figure 4a–c). Tumor burden measured by counting the tumor numbers was significantly higher by overexpression FASN treatment (2.9 ± 1.4) compared with control treatment (1.2 ± 1.0) over an eight week period (Figure 4b), and elevated ascites was found in overexpression FASN treatment mouse compared with control treatment (Figure 4c). Conversely, mouse implanted with reduced FASN SKOV-3 cell by stable knock-down shFASN have fewer tumor numbers (1.4 ± 1.2) compared to control treatment (3.1 ± 2.0) (*p* < 0.05) (Figure 4e), and little ascites was found in the knock-down FASN treatment group (Figure 4f). Taken together, these data suggest a potential involvement of tumor-specific expression of FASN in the regulation of tumor peritoneal implantation *in vivo*.

#### 2.1.5. FASN Leads to Ovarian Cancer Progression of Invasion Ability through Induction of Epithelial-to-Mesenchymal Transition (EMT)

**Figure 5:**
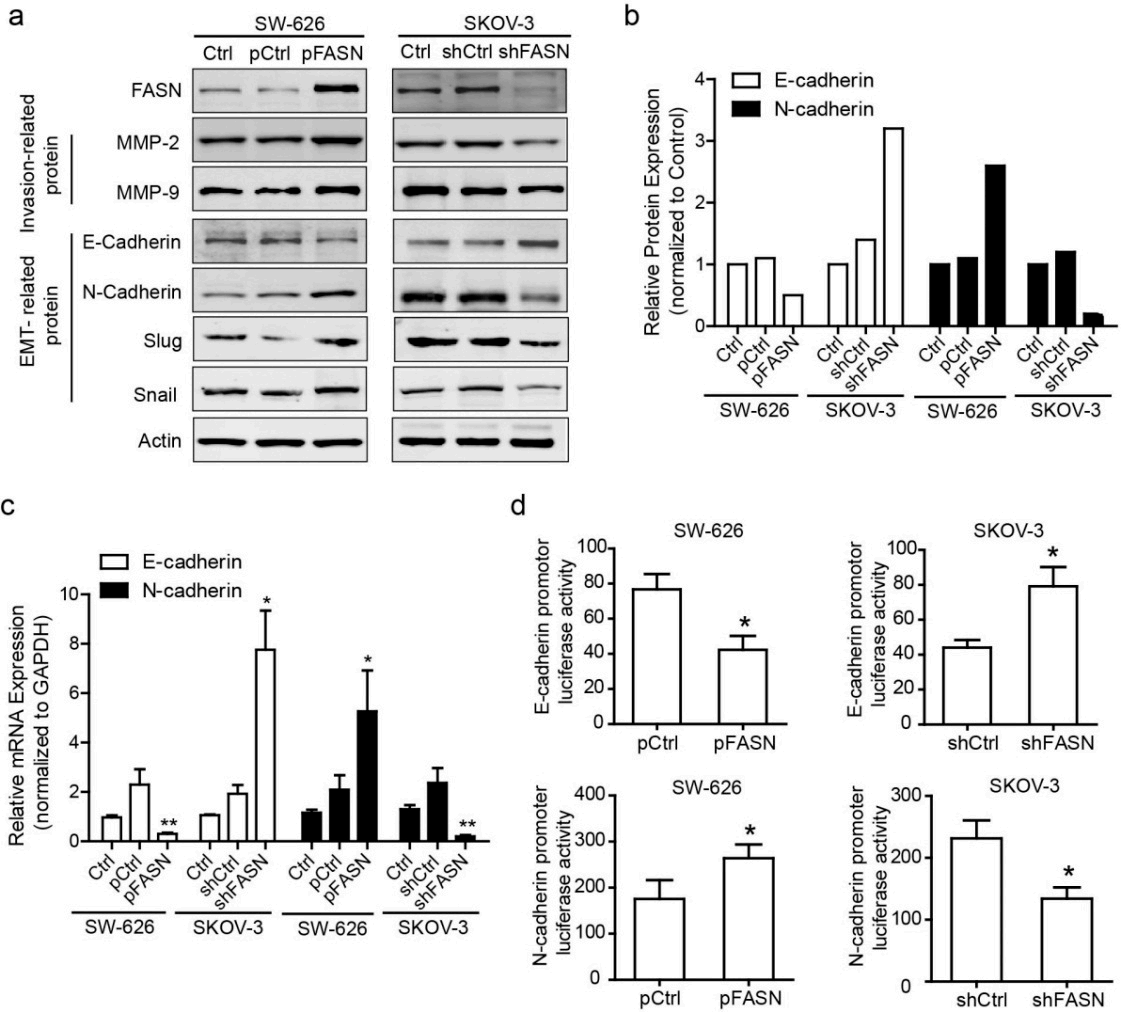
FASN induced epithelial-to-mesenchymal transition (EMT) in ovarian cancer cells. (**a**) Total protein was analyzed by Western blotting. Equal protein loading was confirmed by actin expression; (**b**) Box plots are shown for the relative EMT-related protein in ovarian cancer cells with over-expression or knockdown FASN expression; (**c**) Total RNA was analyzed by quantitative PCR. EMT marker mRNA expression was normalized to GAPDH. Data represent mean ± SEM (*, *p* < 0.05; **, *p* < 0.01); (**d**) The activity of the *E*-cadherin or *N*-cadherin promoter-reporter in ovarian cancer cells with over-expression or knock-down FASN expression is shown. Data represent mean ± SEM (*, *p* < 0.05).

**Figure 6:**
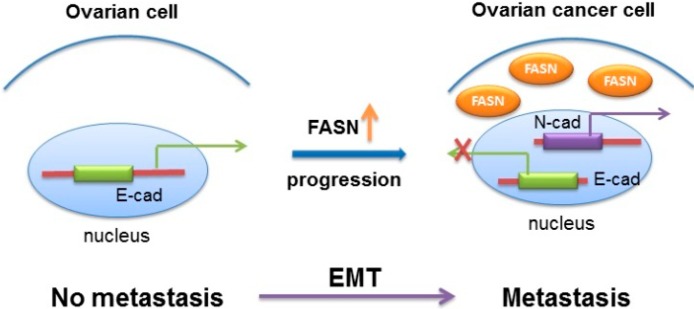
Mechanism of FASN controlling EMT in ovarian cancer. A model is proposed to illustrate upregulated FASN-mediated signaling pathway to engage transcriptional programs leading to EMT, essential for the conversion of ovarian tumors into invasive malignancies.

Invasion and metastasis are considered as associated properties of tumor cells because they use similar processes. To evaluate whether FASN has influence on the invasion-related protein in ovarian cancer cells, we carried out Western blot to detect the expression of MMP2 and MMP9, which can digest various components of extracellular matrix and are crucial to invasion, metastasis and tumorigenesis. Our results showed that overexpression of FASN in ovarian cancer cell line SW-626 enhanced the level of MMP2 and MMP9. Conversely, shRNA knockdown of FASN reduced the level of MMP2 and MMP9 (Figure 5a). To decipher the underlying mechanisms that mediate an increase of metastatic potential of FASN on ovarian cancer cell, we examined the morphology characteristics of FASN stable transfected cells and shFASN stable transfected cells. Interestingly FASN stably transfected cells showed a scattering phenotype with spindle-shaped and fibroblast-like morphology. Conversely, compared with vector stable cells, shFASN stable SKOV-3 cells showed a round morphology and clump growth status. This suggests that the cells may have epithelial-to-mesenchymal transition (EMT). EMT is one of the mechanisms for cancer cells to acquire metastasis and invasion. A key feature of EMT is the switch from *E*-cadherin to *N*-cadherin, cells undergoing EMT downregulate the expression *E*-cadherin accompanied by an increased expression of *N*-cadherin which promotes the interaction with endothelial and stromal components [21]. Snail and Slug are the key proteins for the EMT regulation. We detected the slug and snail expression in protein level when FASN was over-expressed or with knockdown. We found that overexpression of FASN significantly increased the slug and snail expression, while knockdown of FASN decreased the expression of slug and snail (Figure 5a), which indicated that FASN is involved in EMT regulation. In keeping with the morphological changes, our results showed that overexpression of FASN reduced the expression of *E*-cadherin (an epithelial marker), but increased the expression of *N*-cadherin (a mesenchymal cell marker) (Figure 5a). Quantitative results showed that FASN induces EMT related protein in ovarian cancer cells (Figure 5b). Moreover, EMT was confirmed by quantifying epithelial and mesenchymal markers, *E*-cadherin, and *N*-cadherin at mRNA levels (Figure 5c), which suggests the FASN effect on the EMT-related gene at transcriptional level. To verify the hypothesis, we performed a luciferase assay with an *E*-cadherin promoter-luciferase reporter construct and a *N*-cadherin promoter-luciferase reporter construct. As predicted, FASN inhibited the activity of the transfected *E*-cadherin promoter-reporter and enhanced the activity of the transfected *N*-cadherin promoter-reporter (Figure 5d). Apart from the finding, we proposed a model to illustrate the role of FASN-mediated EMT leading to the conversion of ovarian tumors into invasive malignancies (Figure 6).

### 2.2. Discussion

A number of studies have reported the possible involvement of FASN in human carcinogenesis. Our work for the first time examined the influences and the mechanisms of FASN on the metastasis phenotype of ovarian cancer cells *in vitro* and *in vivo*. On the basis of clinical specimen-derived observations, FASN is positively correlated with the peritoneal metastasis. Over-expression of FASN promotes ovarian cancer cell migration *in vitro* and experimental peritoneal metastasis *in vivo*. The oncogenic role of FASN in metastasis is evidenced by a transcriptional regulation of the main components of EMT.

A cancer cell has been recognized as presenting altered metabolism with the observation of increased aerobic glycolysis [22,23]. Fatty acid synthase (FASN) is a key enzyme for the *de novo* synthesis of long-chain fatty acids from acetyl-CoA, malonyl-CoA, and nicotinamide adenine dinucleotide phosphate [24]. More recently, increased expression of FASN has emerged as a malignant phenotype common to numerous human cancers [6,16,25,26,27]. In ovarian cancer, other groups have shown that FASN overexpression is critical to the growth and survival of ovarian carcinomas and FASN interacts with ErbB-systems in ovarian cancer cells [26,28,29,30]. However, the role of FASN in metastasis remains unclear in ovarian cancer. Our study shows that FASN is over-expressed in ovarian cancer, which is consistent with previous reports. Unexpectedly, the expression of FASN does not correlate with the tumor histological stage. In our ovarian cancer tissue array assay, FASN protein expression is strongly associated with ovarian cancer FIGO stage and peritoneal metastasis. This suggests that FASN is involved in the aggression and metastasis of the ovarian cancer cell. An anchorage-independent growth is an important prometastatic characteristic of cancer cells. Our data shows that over-expression of FASN increased the number of colonies, while the number of colonies is markedly reduced in FASN deficient cells. Next, both monolayer wound healing and transwell metastasis assay also proved the pro-metastatic role of FASN in ovarian cancer cell. Actually, FASN has been shown to be involved in the metastasis of colon cancer and oral squamous cell carcinomas [6,15,31]. Nevertheless, different from other carcinomas, ovarian cancer cell frequently spreads from the primary tumor to peritoneal space by ascites and disseminates within the abdominal cavity by direct extension. In advanced high-grade serous carcinomas, extensive seedy of the peritoneal cavity by tumor cells is often associated with ascites [32]. There is often no clearly identifiable precursor lesion, so the events leading to metastatic disease are poorly understood. Our data of *in vivo* experiment shows that FASN regulates peritoneal implantation of ovarian cancer. Over-expression of FASN enhanced the peritoneal implantation and produced more ascites; however, silencing of FASN inhibited the peritoneal colonies growth of ovarian cancer cell and has lower ascites. Therefore, our finding suggested that FASN contributes to ovarian cancer peritoneal metastasis by enhancing cancer cell metastasis.

Ovarian cancer is likely to metastasize via intraperitoneal dissemination and intraperitoneal spread appears to be the culmination of a multi-step process. The complex biochemical pathways involved in this process, however, have only just begun to be clarified [33]. Recent evidence suggests that tumor cells likely participate in the epithelial-mesenchymal transition (EMT), through which tumor cells acquire motility and invasion ability. Emerging evidence suggests that EMT plays a crucial role in the aggressiveness in ovarian cancer including increased migration and invasion ability [34]. We also identified a role for FASN down-regulation of *E*-cadherin, a key regulator of EMT with clinical relevance to aggressive carcinoma. Down-regulation of *E*-cadherin has several important consequences that are of direct relevance to EMT [35]. In ovarian cancer, *E*-cadherin mediated the metastasis and progression [36]. Here we show that over-expression of FASN leads to a reduction in *E*-cadherin, and an increase of *N*-cadherin in ovarian cancer cell lines at both mRNA and protein levels, which indicates that FASN induces EMT in the ovarian cancer cell. In ovarian tissue, *E*-cadherin is regulated at both the mRNA and protein levels in part due to differences in the promoter activation in ovarian carcinoma cells [37]. In dual luciferase assay we show that FASN repressed *E*-cadherin promoter activity and enhanced *N*-cadherin promoter activity. Our data therefore suggests that FASN induces EMT, highlighting the importance of mechanism in ovarian cancer progression and metastasis. However, we also recognize that there are some issues still needed to be done. The detailed mechanism of transcriptional regulation of *E*-cadherin or *N*-cadherin by FASN needs to be further elucidated. A complete understanding of the role of FASN in controlling EMT in tumors is crucial for dissecting the beneficial use of FASN emerging as a potential therapeutic target in ovarian cancer.

## 3. Experimental Section

### 3.1. Patients and Tissue Samples

Ovarian tumors were obtained from a cohort of patients treated at Xinhua Hospital, affiliated with Shanghai Jiaotong University School of Medicine, China, between 2010 and 2013. The median age of the patients was 52.8 years. All patients were diagnosed by pathological analyses based on the International Union Against Cancer (UICC) tumor node metastasis (TNM) stage system. Tissue samples (68) from primary tumors and matched adjacent non-neoplastic ovarian tissues were collected and prepared for tissue microarray (TMA). Fresh-frozen tissues were collected and immediately snap-frozen for protein analyses. The study protocol conformed to the ethical guidelines of the Declaration of Helsinki and was approved by the Ethics Committee of Xinhua Hospital (No. XHEC-D-2010-092; 3 May 2010), Shanghai Jiaotong University School of Medicine. Before inclusion in the study, all patients provided written informed consent.

### 3.2. Cell Culture and Transfection

Human ovarian cancer cell lines SW-626, SKOV-3, and OVCAN3 were obtained from the the American Type Culture Collection (ATCC; Manassas, VA, USA). HO8910, HO8910M were obtained from the Chinese Academy of Sciences Cell Bank of Type Culture Collection (Shanghai, China). The cells were routinely cultured in Dulbecco’s modified Eagle’s medium (DMEM) media supplemented with 10% fetal calf serum, 100 U/mL penicillin and 100 mg/mL streptomycin (Gibco, Grand Island, NY, USA) in 5% CO_2_ at 37 °C. The cells used for our experiments were in the log-phase of growth and were negative for mycoplasma and endotoxin, as confirmed by PCR. SW-626 cells were stably transfected with FASN-overexpression construct (Santa Cruz Biotechnology Inc., Santa Cruz, CA, USA) and control construct, selected using puromycin (Life Technologies, Grand Island, NY, USA), and SKOV-3 cells were stable transfected with shFASN construct (OriGene, Rockville, MD, USA) and control shRNA construct, selected using puromycin (Life Technologies).

### 3.3. Quantitative Real-Time PCR

Quantitative real-time PCR analysis was carried out to detect the mRNA expression of *E*-cadherin and *N*-cadherin. Total RNA extraction from ovarian tissue was performed with Trizol Reagent (Life Technologies). Then, RNA was reverse transcribed and quantified by real-time PCR using the Applied Biosystems 7900 System (Applied Biosystems, Foster City, CA, USA). Sequences of primers used for quantitative Real-time PCR were listed as: *E*-cadherin: forward, 5'-ATTAACCTCACCAATCCTT-3'; reverse, 5'-TTACACCTTGACCTAACG-3'. *N*-cadherin: forward, 5'-GTAGCATAATCACTTGTT-3'; reverse, 5'-GTTACATCCACTTCTATT-3'. Additionally GAPDH was used as an endogenous reference gene (forward, 5'-ATTCCACCCATGGCAAATTC-3'; reverse, 5'-GCATCGCCCCACTTGATT-3').

### 3.4. Immunohistochemical Staining

Standard immunohistochemical procedures were performed using the VECTASTAIN Elite ABC system (Vector Laboratories, Burlingame, CA, USA) according to the manufacturer’s protocol and performed as previously described [38]. Anti-FASN polyclonal antibody (Abcam, Cambridge, MA, USA) was used as primary antibody. The staining intensity (0, no staining; 1, weak staining; 2, moderate staining; and 3, intense staining) and the proportion of stained cells (0, no staining; 1, <10% staining; 2, between 11% and 33% staining; 3, between 34% and 66% staining; and 4, >67% staining) were semiquantitatively determined. The intensity and the percentage of positive cell scores were multiplied (0–12) and classified into three groups: weak (0–4), moderate (5–8) and strong (9–12). All slides were scored by two observers blinded to the pathology and the clinical features.

### 3.5. Western Blot Analysis

Western blot analyses were performed as previously described [38]. Briefly, the cells were lysed and cell extracts were prepared. Cell homogenates were boiled, and then the FASN protein was separated by SDS-PAGE (6%), and others proteins were separated in gel of 12% SDS-PAGE. After overnight incubation with anti-FASN, anti-Slug, anti-snail, anti-MMP2, or anti-MMP9 (Cell Signaling Technology, Beverly, MA, USA), the membranes were incubated with IRDye 800 goat anti-rabbit or IRDye 680 goat anti-mouse secondary antibodies (LI-COR Biosciences, Lincoln, NE, USA). The targeted proteins were detected and quantified on a Li-COR Odyssey infrared imaging system (LI-COR Biosciences).

### 3.6. Plate Colony Formation Assay

For the plate colony formation assay, the cells were plated into a 6-well tissue culture plate (500 cells/well) and incubated at 37 °C for 8 days. The resulting colonies were rinsed with 1× PBS, fixed with methanol for 10 min, and stained with crystal violet. The colonies were photographed and counted for three independent experiments.

### 3.7. Intraperitoneal Xenograft Mouse Model

An intraperitoneal ovarian cancer cell xenograft was used with the approval obtained by the Ethics Committee of Xinhua Hospital (No. XHEC-D-2010-092; 3 May 2010), Shanghai Jiaotong University School of Medicine. Non-obese diabetic/severe combined immunodeficiency (NOD/SCID) mice were purchased from the Shanghai Laboratory Animal Commission of the Chinese Academy of Science, Shanghai, China. NOD/SCID mice at 6 weeks of age were injected intraperitoneally with ovarian cancer cells (1 × 10^6^ cells/0.2 mL). All injection-treated mice were fed for 8 weeks after injection. At the end of 8 weeks, the mice were sacrificed and each tumor burden in the peritoneal cavity was weighed, and the amount of ascites were collected and counted. All experimental use of animals complied with the guidelines of Animal Care at Xinhua Hospital, Shanghai Jiaotong University School of Medicine (Shanghai, China).

### 3.8. Dual Luciferase Reporter Assay

*E*-Cadherin and *N*-cadherin promoter activity was determined using *E*-cadherin or *N*-cadherin luciferase reporter construct (Ecad-Luc or Ncad-Luc), which encoding the human *E*-cadherin 5' promoter (−178 to +92) or human *N*-cadherin 5' promoter (−860 to +20) in pGL3basic (Promega, Madison, WI, USA). Ovarian cancer cells were plated in 24-well plates and transfected with Ecad-Luc or Ncad-Luc and its control plasmid (pRL-TK). At 36 h post transfection, cells were dissolved with passive lysis buffer and luciferase activities were measured using a Dual-Luciferase Reporter Assay System (Promega, Madison, WI, USA) and a microplate luminometer (Promega). The firefly luciferase activities were corrected by the corresponding renilla luciferase activities. Results are representative of three independent experiments.

### 3.9. Statistical Analysis

The data were expressed as the mean ± S.E. of means (S.E.M.). The statistical significance of the difference between two means was assessed using Student’s *t*-test, and the one-way ANOVA with Tukey’s post test was performed for multiple comparisons. All the statistical analyses were performed using GraphPad Prism version 5.0 for Windows (GraphPad Software, San Diego, CA, USA), and statistical significance was set at * *p* < 0.05; ** *p* < 0.01.

## 4. Conclusions

In summary, the authors have provided evidence that higher FASN expression is related to the aggressiveness and peritoneal metastasis of ovarian cancer cells. The authors also propose a probable model by which FASN regulates the transcription of *E*-cadherin/*N*-cadherin and by which FASN-mediated EMT provides a mechanism of escape for the primary cancer cell to disseminate, ensuring cell survival in the peritoneal cavity. These data provide support for the role of FASN inducing EMT and also as a therapeutic target for ovarian cancer.
